# Surpresa! O Que Você é? O Desafio Diagnóstico de uma Massa Cardíaca

**DOI:** 10.36660/abc.20211033

**Published:** 2022-10-05

**Authors:** Mariana Tinoco, Filipa Castro, Sérgio Leite, Francisco Sousa, António Lourenço

**Affiliations:** 1 Hospital da Senhora da Oliveira Guimarães EPE Guimarães Portugal Hospital da Senhora da Oliveira Guimarães EPE , Guimarães – Portugal; 2 Hospital da Luz Guimarães Portugal Hospital da Luz , Guimarães – Portugal

**Keywords:** Massa Cardíaca, Técnicas e Procedimentos Diagnósticos, Diagnóstico por Imagem/métodos, Ecocardiografia Transesofágica/métodos, Ressonância Magnética/métodos, Trombose, Átrios do Coração

**Figura 1:**
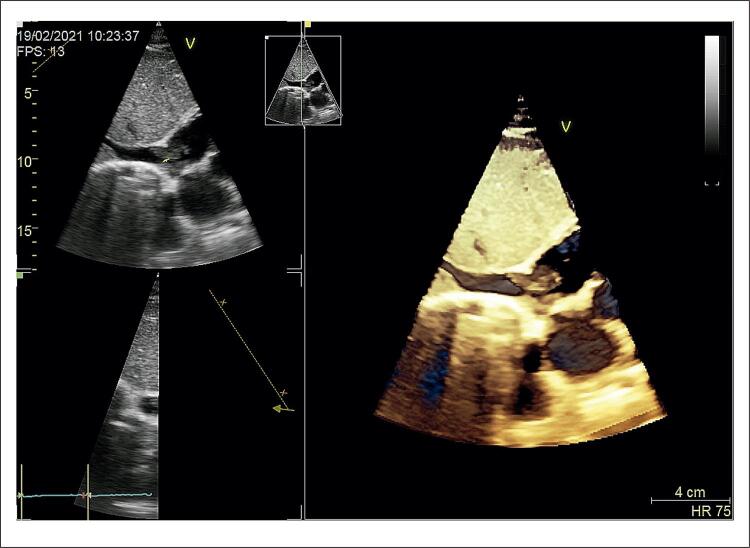
Ecocardiografia transtorácica mostrando a massa atrial direita.

Uma mulher de 49 anos apresentou uma massa atrial direita incidental em uma ecocardiografia transtorácica (ETT) de rotina ( Figura 1 ). Ela não relatou histórico médico significativo anterior, além de uma mamoplastia em 2017 complicada pela ruptura do implante em 2019. Ela não estava em uso de nenhuma medicação, estava em boa forma física e mostrava tolerância total ao exercício. Não havia fatores de risco para tromboembolismo venoso e ela não tinha histórico médico familiar significante. A paciente é ex-tabagista de 12 maços/ano. Ela teve 2 gestações anteriores sem abortos espontâneos. O exame físico foi normal.

O hemograma diferencial, bioquímica geral, eletrocardiograma e radiografia de tórax não mostraram anormalidades.

A angiotomografia de tórax excluiu embolia pulmonar e confirmou uma estrutura hipodensa com morfologia alongada na área de passagem da veia cava inferior (VCI) para o átrio direito, acima da confluência das veias hepáticas, com seu maior eixo medindo cerca de 22mm no plano longitudinal e com espessura aproximada de 5 a 6 mm, de etiologia indeterminada.

A tomografia computadorizada (TC) de crânio foi normal. A TC abdominal e pélvica revelou heterodensidade uterina, identificando pequenas hipodensidades focais infracentimétricas ao redor do endométrio. Não foram encontrados êmbolos sistêmicos nem tumores extracardíacos. A ultrassonografia de membros inferiores excluiu trombose venosa profunda. Ela foi avaliada por um médico ginecologista e foi realizada uma ultrassonografia, que revelou ausência de evidências clínicas ou de imagem de patologias ginecológicas relacionadas ao achado cardíaco. Uma mamografia e ultrassonografia de tireoide não mostraram alterações.

**Figura 2:**
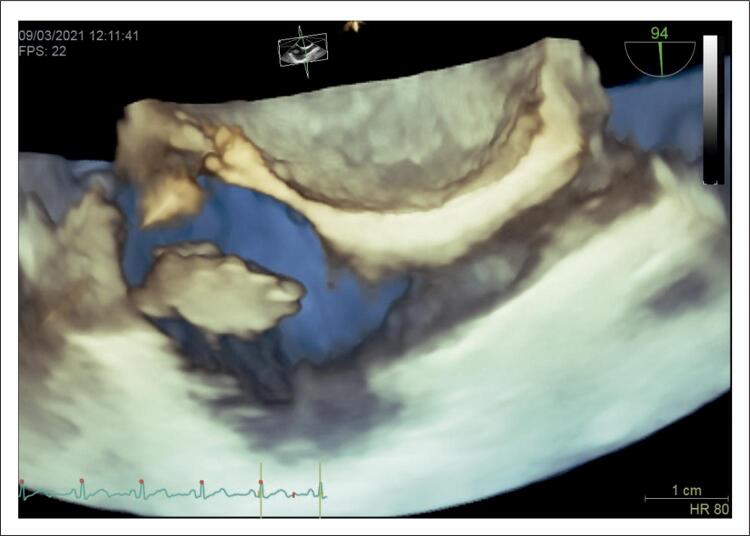
Ecocardiografia transesofágica evidenciando massa atrial direita.

A ecocardiografia transesofágica (ETE) ( Figura 2 ) evidenciou a massa móvel pediculada oriunda da VCI, medindo 29×12mm com contorno muito irregular sem comprometimento hemodinâmico.

O ETE pode levar a informações incertas e esta é a razão pela qual a ressonância magnética (RM) cardíaca foi utilizada sinergicamente com a ecocardiografia. ^1^

**Figura 3:**
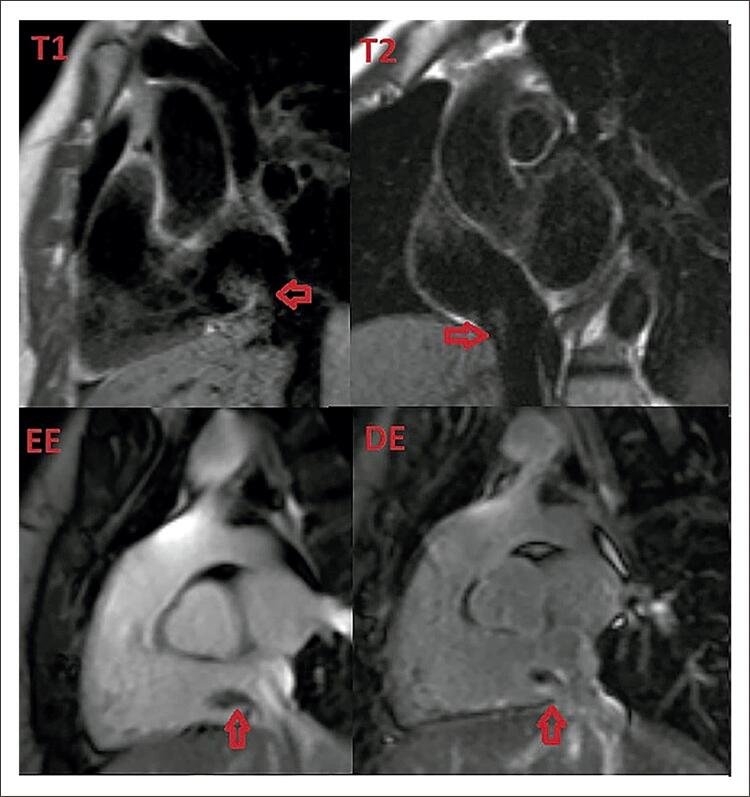
Ressonância magnética cardíaca mostrando a massa atrial direita.

A RM cardíaca ( Figura 3 ) mostrou ventrículos não dilatados com função sistólica global e regional normais, bem como ausência de áreas de infarto, fibrose ou infiltração miocárdica. Também mostrou uma massa muito móvel, de aspecto irregular e vegetante, localizada no interior do átrio direito, adjacente à válvula de Eustáquio, medindo 19x11mm, inserida na parede da VCI/veia supra-hepática através de um pedículo fino. A massa mostrou ser isointensa em relação ao miocárdio nas sequências ponderadas em T2 e levemente hiperintensa nas sequências ponderadas em T1. Nenhuma vascularização aparente foi encontrada na sequência de perfusão de primeira passagem. Após a administração de gadolínio, a massa apresentou realce tardio heterogêneo, mas com ausência de realce precoce.

Sua localização, morfologia e comportamento do sinal sugeriram as hipóteses diagnósticas mais prováveis: mixoma com inserção atípica na VCI, tecido hepático heterotópico, tumor hepatocelular com extensão intracardíaca pela VCI ou leiomiossarcoma da VCI. ^1 , 2^

Nesse ínterim, considerando também a hipótese de trombos intra-atriais, iniciou-se o tratamento com heparina intravenosa (IV) e a massa foi monitorada através de ETT. No entanto, após uma semana de tratamento com heparina IV, o volume da massa não mostrou alterações.

A paciente permaneceu assintomática.

Após consulta com a equipe de Cardiologia, levando em consideração o tamanho da massa e a aparência móvel à ecocardiografia, o que parecia colocar nossa paciente em alto risco para embolia pulmonar, optamos pela exploração cirúrgica com finalidade diagnóstica e curativa.

**Figura 4:**
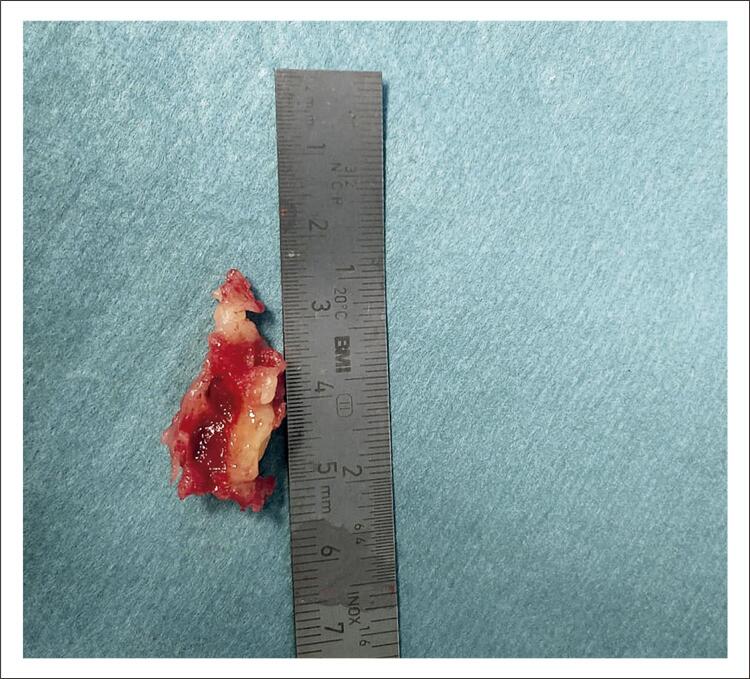
Inspeção cirúrgica da massa cardíaca.

A paciente foi submetida à ressecção cirúrgica da massa, que estava fixada na junção das veias VCI e supra-hepáticas, através de uma esternotomia mediana. A inspeção cirúrgica da massa caracterizou-a como tendo consistência fibroelástica, esbranquiçada e com áreas de aspecto hemorrágico ( Figura 4 ). O exame histológico inesperadamente mostrou apenas material trombótico com várias fases de organização.

A paciente não apresentou complicações pós-operatórias e recebeu alta cinco dias depois com tratamento de anticoagulação oral com apixabana 5 mg, 2 vezes ao dia.

Ela se recuperou bem. Uma ETT realizada três meses após a cirurgia descartou qualquer recidiva de trombo atrial direito.

Uma investigação adicional para o estado trombótico realizado seis meses depois revelou tempos normais de protrombina e tromboplastina parcial ativada, níveis normais de antitrombina III, proteína C e S e homocisteína. A pesquisa de anticorpos anticardiolipina e anticoagulante lúpico foi negativa. A análise genética mostrou estado de homozigose normal para os genes da protrombina e fator V de Leiden. Os níveis de dímero D e fibrinogênio foram normais.

Descrevemos aqui um caso raro de trombo pediculado atrial direito em uma mulher previamente saudável, assintomática, sem cardiopatia estrutural.

Em nossa paciente, as investigações pré-operatórias não conseguiram diferenciar o trombo de um tumor; consequentemente, o diagnóstico foi realizado no pós-operatório.

Apesar das modalidades diagnósticas avançadas e sofisticadas disponíveis, diferenciar massas intracardíacas ainda pode ser um desafio. A apresentação clínica leva à conduta adequada das investigações, sendo a histopatologia uma etapa confirmatória. ^1 , 2^
