# Supplementary material for: Surpresa! O Que Você é? O Desafio Diagnóstico de uma Massa Cardíaca
Source: Arq Bras Cardiol. 2022 Oct 5;119(4):616–8. [Article in Portuguese] doi: 10.36660/abc.20211033 (PMC9563897; doi:10.36660/abc.20211033)
Supplement: Translation

# Translation: Peek-A-Boo What are You? The Diagnostic Challenge of a Cardiac Mass

**Authors:** Mariana Tinoco, Filipa Castro, Sérgio Leite, Francisco Sousa, António Lourenço

**Affiliations:** 1 Portugal Hospital da Senhora da Oliveira Guimarães EPE, Guimarães – Portugal; 2 Portugal Hospital da Luz, Guimarães – Portugal

**Keywords:** Cardiac Mass, Diagnostic Techniques and Procedures, Diagnostic, Imaging/methods, Echocardiography Transesophageal/methods, Resonance Magnetic/methods, Trombosis, Heart Atrial

A 49-year-old female was found to have an incidental right atrial mass on a routine transthoracic echocardiography (TTE) ( Figure 1 ). She reported no previous significant medical history, besides a mammoplasty in 2017 complicated with implant rupture in 2019. She did not take any medication, was fit and well, and showed full exercise tolerance. There were no risk factors for venous thromboembolism, and she had no significant family medical history. The patient is a former smoker of 12 pack-years. She had 2 previous pregnancies with no miscarriages. The physical examination was unremarkable.

**Figure 1:** Transthoracic echocardiography showing the right atrial mass.

The differential blood count, general biochemistry, electrocardiogram, and chest radiograph showed no abnormalities.

A CT angiography of the thorax excluded pulmonary embolism and confirmed a hypodense structure with elongated morphology in the area of passage from the inferior vena cava (IVC) to the right atrium, above the confluence of the hepatic veins, with the longest axis measuring about 22mm in the longitudinal plane, with an approximate thickness of 5 to 6 mm, of undetermined etiology.

The cranial computed tomography (CT) scan was normal. The abdominal and pelvic CT scans revealed uterine heterodensity, identifying small infracentimetric focal hypodensities surrounding the endometrium. No systemic emboli or extracardiac tumours were found. A lower-limb ultrasonography excluded deep vein thrombosis. She was assessed by a gynecologist and an ultrasound was performed, which revealed absence of clinical or imaging evidence of gynecological pathologies related to the cardiac finding. A mammography and thyroid ultrasonography were unremarkable.

The transesophageal echocardiography (TEE) ( Figure 2 ) showed a mobile pedunculated mass arising from the IVC, which measured 29×12mm and showed a very irregular contour, without entailing hemodynamic compromise.

**Figure 2:** Transesophageal echocardiography showing the right atrial mass.

The TEE may lead to uncertain information and this is the reason why a cardiac MRI was used synergistically with the echocardiography.^1^

A cardiac magnetic resonance imaging (MRI) ( Figure 3 ) showed non-dilated ventricles, with normal global and regional systolic function, as well as absence of areas of infarction, fibrosis or myocardial infiltration. It also showed a very mobile mass, with an irregular and vegetative appearance located inside the right atrium, adjacent to the Eustachian valve, measuring 19x11mm, attached to the wall of the IVC/supra-hepatic vein through a thin pedicle. The mass showed to be isointense relative to the myocardium on T2-weighted sequences and slightly hyperintense on T1-weighted sequences. No apparent vascularization was found in the first-pass perfusion sequence. After gadolinium administration, the mass showed a heterogeneous late enhancement but with absence of early enhancement.

**Figure 3:** Cardiac magnetic resonance imaging showing the right atrial mass.

Its location, morphology and signal behavior suggested the most likely diagnostic hypotheses: myxoma with atypical insertion in the IVC, heterotopic liver tissue, hepatocellular tumor with intracardiac extension through the IVC or IVC leiomyosarcoma.^1 , 2^

In the meantime, also considering the hypothesis of intra-atrial thrombi, the treatment with intravenous (IV) heparin was initiated and the mass was monitored through TTE. However, one week after IV heparin treatment, the mass volume did not change.

The patient remained asymptomatic.

After consultation with the Heart Team, taking into account the size of the mass and the mobile appearance on the echocardiography that seemed to place our patient at high risk for pulmonary embolism, the case assessment led us to choose surgical exploration with a diagnostic and curative purpose.

The patient underwent surgical resection of the mass, which was attached at the junction of the IVC and suprahepatic veins, through a median sternotomy. The surgical inspection of the mass characterized it as having a fibroelastic consistency, whitish and with areas of hemorrhagic aspect ( Figure 4 ). The histological examination unexpectedly showed only thrombotic material with several phases of organization.

**Figure 4:** Surgical inspection of the cardiac mass.

The patient had no postoperative complications and was discharged 5 days later with oral anticoagulation treatment with apixaban 5 mg twice a day.

She recovered well. A TTE performed 3 months after the surgery ruled out any relapse of a right atrial thrombus.

A further workup for thrombotic state performed 6 months later revealed normal prothrombin and activated partial thromboplastin times, normal antithrombin III, protein C and S and homocysteine levels. Screening for anticardiolipin antibodies and lupus anticoagulant was negative. The genetic analysis showed normal homozygous state for prothrombin and factor V Leiden genes. D-dimer and fibrinogen levels were normal.

We describe here a case of a rare right atrial pedunculated thrombus in a previously healthy, asymptomatic woman without structural heart disease.

In our patient, the preoperative investigations could not differentiate the thrombus from a tumor; consequently, the diagnosis was made postoperatively.

Despite the available advanced and sophisticated diagnostic modalities, differentiating intracardiac masses can still be challenging. Clinical presentation leads to the appropriate conduit of investigations, and histopathology is a confirmatory step.^1 , 2^
